# Routine clinical practice in the periprocedural management of edoxaban therapy is associated with low risk of bleeding and thromboembolic complications: The prospective, observational, and multinational EMIT‐AF/VTE study

**DOI:** 10.1002/clc.23379

**Published:** 2020-05-14

**Authors:** Paolo Colonna, Christian von Heymann, Amparo Santamaria, Manish Saxena, Thomas Vanassche, Diana Wolpert, Petra Laeis, Robert Wilkins, Cathy Chen, Martin Unverdorben

**Affiliations:** ^1^ Polyclinic of Bari—Hospital, Department of Cardiology Bari Italy; ^2^ Vivantes Klinikum im Friedrichshain, Department of Anaesthesia & Intensive Care Medicine Emergency Medicine, and Pain Therapy Berlin Germany; ^3^ Hematology Department, Alicante University Hospitals University Vinalopó Salut and Torrevieja Salut Alicante Spain; ^4^ William Harvey Research Institute Barts Health NHS Trust, Charterhouse Square London UK; ^5^ Department of Cardiovascular Sciences University Hospitals (UZ) Leuven Leuven Belgium; ^6^ Daiichi Sankyo Medical Affairs Europe Munich Germany; ^7^ QPS Consulting, LLC Ashburn Virginia USA; ^8^ Daiichi Sankyo Inc., Global Medical Affairs Specialty and Value Products Basking Ridge New Jersey USA

**Keywords:** atrial fibrillation, bleeding, edoxaban, NOAC, periprocedural management, venous thromboembolism

## Abstract

**Background:**

Guidance for periprocedural anticoagulant management is mainly based on limited data from Phase III or observational studies and expert opinion.

**Hypothesis:**

EMIT‐AF/VTE was designed to document the risks of bleeding and thromboembolic events in more than 1000 patients on edoxaban undergoing diagnostic and therapeutic procedures in clinical practice.

**Methods:**

Routine care in a multinational multicenter, prospective observational study. Participants were adult patients with atrial fibrillation and/or venous thromboembolism treated with edoxaban for stroke prevention or for secondary prevention in venous thromboembolic disease, undergoing a wide range of diagnostic and therapeutic procedures. Edoxaban therapy was interrupted periprocedurally at the treating physician's discretion. Patients were evaluated from 5 days pre‐ until 30 days postprocedure. Primary outcome was the incidence of International Society on Thrombosis and Haemostasis defined major bleeding; secondary outcomes included incidence of clinically relevant non‐major bleeding, acute coronary syndrome, and acute thromboembolic events.

**Results:**

Outcomes and management are reported for the first procedures in 1155 unselected patients. Five cases of major bleeding (0.4%) and eight of clinically relevant non‐major bleeding (0.7%) were documented, five (38%) of which occurred outside the period of likely edoxaban effect (last edoxaban dose ≥3 days prior to bleeding). Five (0.4%) deaths from any cause, seven acute thromboembolic events (0.6%) including two cardiac deaths (0.2%) in six patients, and one acute coronary event (0.1%) occurred.

**Conclusions:**

The periprocedural bleeding and acute thromboembolic event risks for patients treated with edoxaban were low. This can help inform both clinical routine and guidelines for the periprocedural management of edoxaban.

## 1 INTRODUCTION

Non‐vitamin K dependent oral anticoagulants (NOAC) have become the preferred and recommended long‐term therapy^1^, ^2^ for stroke prevention in patients with atrial fibrillation (AF) and for the treatment and secondary prevention of venous thromboembolism (VTE). Compared to vitamin K antagonists (VKA), NOACs exhibit a rapid onset of action and a shorter effect duration, so a shorter interruption, if any, prior to invasive procedures might be expected. At least 10% of these patients require diagnostic and therapeutic procedures each year.^3^ Guidance for clinicians is mainly based on pharmacokinetic data, Phase III studies involving cardiovascular procedures such as PCI and AF ablation,^4^, ^5^, ^6^ expert opinion or guidelines (eg, from the European Heart Rhythm Association [EHRA]^7^ or other specialist societies^8^, ^9^) and practical questions remain.

The periprocedural management of NOAC treatment focuses on reducing the risk of bleeding, without complicating the procedure with an increased risk of thromboembolic events. Current evidence suggests that if the bleeding risk is low, interruption of anticoagulation therapy may not be necessary.^10^ However, this has mainly been studied in patients treated with VKA.

A recently published^11^ prospective study using a predefined periprocedural management protocol for apixaban, dabigatran and rivaroxaban, found event rates for major bleeding (MB) at 30 days lower than the prespecified 2% for all three NOACs, whereas a different strategy resulted in low major bleeding rates in patients treated with dabigatran.

Previous studies with edoxaban have demonstrated noninferiority to warfarin for stroke prevention in AF and for VTE treatment/secondary prevention of thromboembolic events and shown a reduced MB or clinically relevant non‐major bleeding (CRNMB) incidence, and in particular a lower incidence of potentially life‐threatening intracranial bleeding.^12^, ^13^ Currently, however, periprocedural management data for edoxaban are only available from post hoc analyses of recent studies^14^, ^15^Important questions remain on time and modalities of NOAC interruption, as there is minimal information to decide if current recommendations provide the optimal balance of bleeding and thrombo‐embolic risk.^16^

This first prospective study was designed to collect information about the periprocedural management of patients receiving edoxaban, and to document bleeding and thromboembolic events in a large unselected sample of more than 1000 patients on long‐term edoxaban therapy.

## 2 METHODS

A description of the objectives and design of the study has been previously published.^17^ The study was a multicenter, prospective, observational, study conducted in Europe (NCT02950168) in accordance with the Declaration of Helsinki and with local IRB approvals. Written informed consent was obtained from participants prior to enrolment. The periprocedural management of anticoagulant therapy was at the discretion of the investigator, including any decision whether to interrupt edoxaban therapy and the timing/duration of any interruption of edoxaban treatment. The site investigator determined the edoxaban patient management approach based upon individual patient findings, in the context of published guidelines and professional experience: no attempt was made to influence patient management by the study authors, study team or the sponsor.

### 2.1 Patient recruitment

Enrolment commenced in December 2016 and was complete in July 2018. Patients were recruited from 326 centers from Belgium, Germany, Italy, Netherlands, Portugal, Spain, and the United Kingdom. Eligible patients were ≥18 years of age, had AF or VTE, were treated with edoxaban according to the local labels, were not enrolled in any other study concurrently, and underwent any type of diagnostic or therapeutic procedure. Data from the first procedure in each patient is reported.

### 2.2 Observations

The observation period of the study started 5 days before the procedure and ended 30 days afterwards. To enhance data capture, patients received a memory aid booklet at enrolment into the study, which was reviewed at the end of the study. Date of visit, details of edoxaban treatment, and clinical outcomes were documented at 30 days after each procedure. No interruption of edoxaban therapy was defined if edoxaban was administered on each day of the observation period including at any time on the day of the procedure. Any interruption of edoxaban treatment was recorded as the number of days without administration of edoxaban, preprocedural and/or postprocedural. Any dose skipped before or on the day of the procedure was defined as preprocedural. Day one of follow‐up was defined as the day of procedure.

The primary outcome was the incidence of MB using the International Society of Thrombosis and Haemostasis (ISTH) definition.^18^ Secondary outcomes included the incidences of CRNMB and all‐cause mortality. CRNMB events were defined as overt bleeding that required medical attention but that did not fulfill the criteria for MB. Other secondary outcomes of the study were evaluation of the periprocedural interruption, dosing of edoxaban, incidence of acute coronary syndrome (ACS) and acute thromboembolic events (ATE) (a composite of non‐hemorrhagic stroke, transient ischemic attack [TIA], systemic embolic events [SEE], deep vein thrombosis [DVT], and pulmonary embolism [PE]. All incidents of MB, CRNMB, ACS and ATE were reviewed and unanimously adjudicated by the Steering Committee. Bleeding had to commence during or after the procedure to be classified as a procedural complication.

Concomitant medications, periprocedural EHRA bleeding risk^7^, HAS‐BLED^19^ (hypertension, abnormal renal/liver function, stroke, bleeding history or predisposition, labile international normalized ratio, elderly, drugs/alcohol concomitantly) score, CHA_2_DS_2_‐VASc (congestive heart failure, hypertension, age ≥75 [doubled], diabetes, stroke [doubled]‐vascular disease, age 65‐74 years, and gender [female]) score, details of edoxaban treatment, diagnostic/therapeutic procedures, and clinical findings were documented at baseline and during the periprocedural period.

### 2.3 Statistical analysis

The study planned to document about 1415 procedures. This estimate was based on an assumed incidence of MB at 30 days of 1.5%, based on published data from the Dresden NOAC registry^20^ which reported a 1.2% MB incidence during a 30 ± 5 day postprocedural follow up period, and another cohort study which identified a 1.8% MB incidence during a 30‐day follow‐up.^21^ Assuming a 5% dropout rate, 1415 procedures were expected to allow for a reasonable estimation of the incidence of MB, with a precision of the 95% confidence interval of the event rate of 0.65%.

All clinical data was documented using the Medidata Rave Electronic Data Capture system (Medidata Solutions Inc., New York, New York). Binary, categorical, and ordinal parameters were summarized by means of absolute and percentage numbers. Numerical data were described by standard statistics. The statistical analyses were performed using SAS version 9.4 (SAS Institute, Cary, North Carolina). All analyses were descriptive and exploratory.

## 3 RESULTS

**FIGURE 1:**
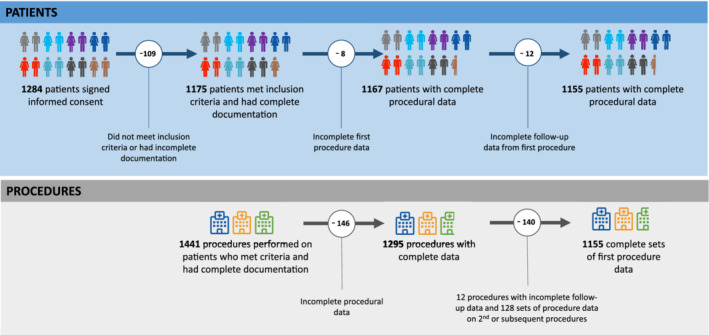
Dataset definition and patient exclusions

Informed consent for the EMIT‐AF/VTE study was obtained from 1284 subjects, from whom data on 1441 procedures was collected. In 109 patients early enrolment followed by delay or cancellation of the index procedure led to missing inclusion criteria or incomplete documentation. Therefore, there were 120 procedures less than the planned 1415; this reduction in numbers of procedures changed the precision of the 95% confidence interval of the event rate from 0.65% to 0.70%. The evaluable data set contains complete subject baseline and complete periprocedural data from the first procedure for 1155 patients. Of 1155 first procedures, 1100 (95.2%) were prescheduled, 50 (4.3%) unplanned (emergency/urgent), and 5 (0.4%) unknown. One hundred and twenty‐eight second or subsequent procedures were performed in these 1155 patients; data from these procedures will be reported separately. The derivation of this data set is summarized in Figure 1.

Four hundred and thirty‐four procedures were diagnostic or interventional transcatheter vascular procedures; 136 cardiothoracic and vascular procedures; 127 dentistry procedures; 117 gastroenterology procedures. Figure S1 details all procedural groups, including those procedural groups that were less than 10% of the total number of procedures, and the number of complications in each procedural group.

### 3.1 Patient and procedure characteristics

In line with studies on other NOACs,^10^, ^20^ the patients reported here were elderly (45% ≥ 75 years of age), over‐weight (body mass index [BMI] 28.1 ± 5.0 kg/m^2^) with expected cardiovascular co‐morbidities such as hypertension (71%), diabetes mellitus (22%), and coronary artery disease (21%). Nineteen percent of patients had a creatinine clearance ≤ 50 mL/min, the threshold for edoxaban dose reduction. Demographic, medical history, medication, and other baseline data are summarized in Table 1.

**TABLE 1:** Clinical characteristics and patient data: all subjects and grouped by EHRA procedural bleeding risk

	All subjects	Minor risk	Low risk	High risk
Characteristic	N = 1155	N = 294	N = 581	N = 280
*Demographics*				
Age at enrolment	71.9 ± 10.4	73.4 ± 10.0	71.0 ± 10.8	72.4 ± 9.8
Age < 65 years	233 (20.2%)	43 (14.6%)	137 (23.6%)	53 (18.9%)
≥65 to <75 years	401 (34.7%)	98 (33.3%)	202 (34.8%)	101 (36.1%)
≥75 years	521 (45.1%)	153 (52.0%)	242 (41.7%)	126 (45.0%)
Male	719 (62.3%)	169 (57.5%)	376 (64.7%)	174 (62.1%)
Female	436 (37.7%)	125 (42.5%)	205 (35.3%)	106 (37.9%)
Body weight ≤ 60 kg	86 (7.5%)	22 (7.5%)	31 (5.3%)	33 (11.8%)
BMI kg/m^2^	1122, 28.13 ± 4.99	279, 28.11 ± 4.95	573, 28.30 ± 5.10	270, 27.81 ± 4.81
*Indication for Edoxaban (total is greater than 1155 because 23 patients had both AF and VTE, and in 9 patients the indication was not recorded)*				
AF	1070 (92.6%)	247 (84.0%)	564 (97.1%)	259 (92.5%)
Paroxysmal	540 (50.2%)	119 (45.6%)	294 (51.9%)	127 (51.2%)
Persistent	257 (23.9%)	56 (21.5%)	154 (27.2%)	47 (19%)
Long‐standing persistent	38 (3.5%)	23 (4.1%)	23 (4.1%)	4 (1.6%)
Permanent	238 (22.1%)	93 (16.4%)	96 (16.4%)	70 (28.2%)
Missing	3	3		
VTE	99 (8.6%)	36 (12.2%)	27 (4.6%)	36 (12.9%)
DVT only	63 (5.5%)	27 (9.2%)	12 (2.1%)	24 (8.6%)
PE	36 (3.1%)	9 (3.1%)	15 (2.6%)	12 (4.3%)
*Medical history (% based on all patients in sub‐group)*				
Hypertension	821 (71.1%)	220 (74.8%)	400 (68.8%)	201 (71.8%)
Dyslipidaemia	471 (40.8%)	113 (38.4%)	232 (39.9%)	126 (45.0%)
Diabetes mellitus	256 (22.2%)	62 (21.2%)	138 (23.8%)	56 (20.0%)
Coronary heart disease	247 (21.4%)	53 (18.0%)	147 (25.3%)	47 (16.8%)
Valvular heart disease	212 (18.4%)	29 (9.9%)	133 (22.9%)	50 (17.9%)
Renal disease	163 (14.1%)	30 (10.2%)	92 (15.8%)	41 (14.6%)
Congestive heart failure	149 (12.9%)	24 (8.2%)	92 (15.8%)	33 (11.8%)
Malignancies	122 (10.6%)	30 (10.2%)	45 (7.7%)	47 (16.8%)
*Renal function*				
Creatinine clearance mL/min	1023; 77.3 ± 32.5	240; 74.4 ± 33.2	535; 78.5 ± 33.8	248; 77.5 ± 28.6
Creatinine clearance ≤50 mL/min (% based on in group above)	190 (18.6%)	43 (17.9%)	104 (19.4%)	43 (17.3%)
*Concomitant medications (taken in the 5 days prior to procedure)*				
Oral NSAIDs	13 (1.1%)	3 (1.0%)	6 (1.0%)	4 (1.4%)
Proton pump inhibitors	196 (17.0%)	46 (15.6%)	96 (16.5%)	54 (19.3%)
Heparin (including LMWH)	143 (12.4%)	11 (3.7%)	65 (11.2%)	67 (23.9%)
ASA	49 (4.2%)	4 (1.4%)	33 (5.7%)	12 (4.3%)
Clopidogrel	26 (2.3%)	1 (0.3%)	21 (3.6%)	4 (1.4%)
Ticagrelor	1 (0.1%)	0	1 (0.2%)	0
ASA + Clopidogrel	8 (0.7%)	0	7 (1.2%)	1 (0.4%)
Ciclosporin, dronedarone, erythromycin, or ketoconazole	4 (0.4%)	0	1 (0.2%)	3 (1.1%)
Others P‐gp inhibitors or inducers	16 (1.4%)	7 (2.4%)	6 (1.0%)	3 (1.1%)
Contraceptives or post‐menopausal therapy	15 (1.3%)	3 (1.0%)	6 (1.0%)	6 (2.1%)
*Risk scores*				
HAS‐BLED score	724; 1.7 ± 1.1	195; 1.5 ± 0.9	344; 1.7 ± 1.1	185; 1.9 ± 1.1
>3	42 (5.8%)	4 (2.1%)	23 (6.7%)	15 (8.1%)
CHA_2_DS_2_‐VASc score	1115; 3.2 ± 1.6	281; 3.4 ± 1.4	563; 3.0 ± 1.6	271; 3.4 ± 1.7
>3	463 (41.5%)	125 (44.5%)	215 (38.2%)	123 (45.4%)
*Edoxaban dose*				
30 mg/day	222 (19.2%)	57 (19.4%)	99 (17.0%)	66 (23.6%)
60 mg/day	933 (80.8%)	237 (80.6%)	482 (83.0%)	214 (76.7%)
Missing	47 (4.1%)	9 (3.1%)	22 (3.8%)	16 (5.7%)
*Edoxaban dosage*				
30 mg/day total	233 (20.2%)	62 (21.1%)	101 (17.4%)	70 (25.0%)
30 mg/day appropriate	114 (9.9%)	26 (8.8%)	49 (8.4%)	39 (13.9%)
30 mg/day underdosed	110 (9.5%)	34 (11.6%)	47 (8.1%)	29 (10.4%)
30 mg/day unknown	9 (0.8%)	2 (0.7%)	5 (0.9%)	2 (0.7%)
60 mg/day total	875 (75.8%)	223 (75.9%)	458 (78.8%)	194 (69.3%)
60 mg/day appropriate	763 (66.1%)	193 (65.7%)	403 (69.4%)	167 (59.6%)
60 mg/day overdosed	81 (7.0%)	17 (5.8%)	46 (7.9%)	18 (6.4%)
60 mg/day unknown	31 (2.7%)	13 (4.4%)	9 (1.6%)	9 (3.2%)

*Note:* All data presented as mean ± SD; number (%); or number, mean ± SD.

Abbreviations: AF, atrial fibrillation; BMI, body mass index; CHA_2_DS_2_‐VASc, congestive heart failure, hypertension, age ≥ 75 [doubled], diabetes, stroke [doubled]‐vascular disease scores; EHRA, European Heart Rhythm Association; HAS‐BLED, hypertension, abnormal renal/liver function, stroke, bleeding history or predisposition, labile international normalized ratio, elderly, drugs/alcohol concomitantly; LMWH, low molecular weight heparin; NSAID, nonsteroidal anti‐inflammatory drug; P‐gp, P glycoprotein; VTE, venous thrombo‐embolism.

### 3.2 Bleeding and thromboembolic outcomes and risk prediction

In 1155 procedures, there were 49 (4.2%; 95% CI 3.2‐5.6%) episodes of bleeding, of which five (0.4%, 95% CI 0.1‐1.0%) were classified as MB and eight (0.7%, 95% CI 0.3‐1.4%) as CRNMB. There were five (0.4%, 95% CI 0.1‐1%) deaths from any cause. Risk of any bleeding was greater with increasing EHRA predicted risk (minor 3.1% to high 5.7%), HAS‐BLED score (low 2.7% to high 4.0%) and CHA_2_DS_2_‐VASc score (low 3.8% to high 4.5%). Risk of MB/CRNMB also increased with higher EHRA and HAS‐BLED scores (Table S1).

There were six (0.5%, 95% CI 0.2‐1.1%) ATE (including one ACS) and two (0.2%, 95% CI 0‐0.6%) cardiovascular deaths (including one death due to stroke) in seven patients. ATE/ACS were confined to the moderate and high risk CHA_2_DS_2_‐VASc groups. Risk stratification and associated outcomes are summarized in Table S1. Table 2 provides details of the 13 patients with MB/CRNMB, seven with ATE/ACS/cardiac death, and three patients with non‐cardiac deaths.

**TABLE 2:** Case details of all subjects with MB/CRNMB, ACS/ATE, or death (for any reason) as an outcome

Case #	Procedure type	Procedure description	Age/gender/dose	MB/ATE/death	Day of event relative to procedure (day 1)	EHRA risk group	Pre‐/post‐procedural interruption (d)	Outcome	Scheduled or emergency. Heparin use	Medications
1	Endoscopy: colonoscopy and polypectomy	Patient admitted due to life‐threatening bleeding. Final diagnosis: colorectal polyp bleeding.	65/female 60 mg	MB	1	Minor	1/29	Recovered	Scheduled No heparin use	PGP, PPI
2	Vascular access and transcatheter diagnostics and interventions	Coronary angiography (femoral access)	68/male 60 mg	CRNMB	6	Low	1/1	Recovered	Scheduled No heparin use	PPI
3	Urology	Transurethral resection of bladder tumor (TURBT)	68/male 30 mg	CRNMB	5	High	0/0	Recovered	Scheduled Start of heparin 4 days after event for whole follow‐up	LMWH
4	Ear, nose, throat	NA	73/female 60 mg	CRNMB	14	Low	0/0, stop of edoxaban 1 day after bleeding, restart 1 week after bleeding	Recovered	Scheduled Heparin from day of procedure until 1 week after bleeding	LMWH
5	Vascular access and transcatheter diagnostics and interventions	Transfemoral aortic valve replacement. After procedure: Hb‐relevantgastrointestinal‐bleeding related to Mallory–Weiss syndrome	80/male 60 mg	MB	2	Low	1/0 (last edoxaban on day of event)	Recovered	Scheduled Heparin: 1 day before bleeding on day of procedure	Clopidogrel, UFH
6	Orthopedic	Femoral fracture reduction and synthesis	77/female 30 mg	MB	1	High	3/8	Recovering	Unplanned/emergency Heparin: 1 day before procedure until restart of edoxaban	LMWH, PPI
7	Orthopedic	Partial hip replacement	82/female 60 mg	MB	4	Low	3/no restart date provided	Recovering	Scheduled Heparin for 3 days prior to procedure and ongoing during follow‐up	LMWH, PPI
8	Gastroenterology	Colonoscopy ± polypectomy	72/female 60 mg	CRNMB	22	High	2/0 stop of edoxaban 2 days prior to event, restart 1 day after event	Recovered	Scheduled No heparin use	
9	Vascular access and transcatheter diagnostics and interventions	Electrophysiologic interventions	66/female 60 mg	CRNMB	1	Low	4/1	Recovered	Scheduled Heparin from 1 day prior to 1 day after procedure	LMWH
10	Gastroenterology	Colonoscopy ± polypectomy	68/male 60 mg	CRNMB	1	High	0/0	Recovered	Unplanned/emergency No heparin use	
11	Cardiothoracic and vascular surgery	Insertion of pacemaker/defibrillator	78/male 30 mg	CRNMB	30	Low	2/0, last dose 6 days before bleeding	Recovering	Scheduled Heparin from 5 days prior to bleeding until end of follow‐up	LMWH
12	Gynecology	Hysterectomy ± uterine polypectomy	63/female 60 mg	CRNMB	18	High	2/27	Recovered	Scheduled Heparin from 1 day prior to procedure until 10 days after event	LMWH
13	Vascular access and transcatheter diagnostics and interventions	Computed tomography guided radiofrequency ablation	45/male 60 mg	MB	3	High	2/0 Edoxaban on day of event, restart 6 days after event	No data available	Scheduled Heparin from day of event until restart edoxaban	LMWH, PPI
14	Cardiothoracic and vascular surgery	Pericardiocentesis	54/male 60 mg	Ischemic stroke & CV death	3/3	High	4/unknown because of death	Death	Scheduled Heparin from stop of edoxaban until event	LMWH
15	Vascular access and transcatheter diagnostics and interventions	PCI	80/male 60 mg	TIA	1	Low	2/0	Recovered	Scheduled No heparin use	ASA
16	Surgery (general)	Other open intra‐abdominal surgery	83/female 60 mg	SE	16	High	4/20 restart 4 days after event	Recovered	Unplanned/emergency Heparin during edoxaban interruption	
17	Surgery (general)	Other open intra‐abdominal surgery	61/male 60 mg	PE/VTE	20	High	5/29, no restart of edoxaban during follow‐up	Recovering	Scheduled Heparin from edoxaban stop ongoing during follow‐up	LMWH, NSAID
18	Orthopedic	Total hip replacement/arthroplasty	76/male 60 mg	PE/VTE	13	High	2/16	Recovered with sequelae	Scheduled Heparin during edoxaban interruption	LMWH, UFH
19	Laryngoscopy/esophagoscopy (direct)	Non‐ST elevation myocardial infarction	65/male 30 mg	ACS	2	Minor	1/restart edoxaban 1 day after event	Recovered	Scheduled Heparin from stop of edoxaban until end of follow‐up	ASA, UFH
20	Coronary angiography (femoral access)	CV death	73/male 60 mg	CV death	21	Low	1/10	Death	Scheduled No heparin use	ASA
21	Conservative treatment of bone fractures/torn ligaments	Cardiorespiratory insufficiency	89/female 30 mg	Death not CV related	3	High	5/unknown because of death	Not applicable	Unplanned/emergency Heparin from stop of edoxaban ongoing	LMWH, PPI
22	Coronary angiography (radial access)	Unknown reason for death	66/male 60 mg	Death not CV related	20	Low	No interruption	Not applicable	Scheduled No heparin use	
23	Endoscopy	Other cause (eg, infection/suicide/accidental or trauma/hepatobiliary/renal/other)	74/male 60 mg	Death not CV related	13	Minor	0/12, stop edoxaban 11 days before death	Not applicable	Scheduled No heparin use	

Abbreviations: ACS, acute coronary syndrome; ASA, acetylsalicylic acid; ATE, acute thromboembolic event; CRNMB, clinically relevant non‐major bleeding; CV, cardiovascular; EHRA, European Heart Rhythm Association; LMWH, low molecular weight heparin; MB, major bleeding; NSAID, non‐steroidalanti‐inflammatory drug; P‐gp, P glycoprotein; PPI, proton pump inhibitor; UFH, unfractionated heparin.

Of the 1068 patients with complete data to determine the appropriate labeled dose, 877 (82.1%) received the recommended edoxaban dose based upon label requirements and recorded nine episodes of MB or CRNMB (1.0%). One hundred and ten patients (10.3%) were on non‐recommended 30 mg dosing, with one episode of MB or CRNMB (0.9%), and 81 patients (7.6%) were on non‐recommended 60 mg dosing, with three episodes of MB or CRNMB (3.7%). There were five ATE and three deaths in the 877 patients receiving recommended edoxaban doses (0.9%), and one ATE, one ACS and two deaths in patients receiving a non‐recommended dose (2.1%; 2.5% in those receiving a greater than recommended dose, and 1.8% in those receiving a smaller dose than recommended). Of the 195 patients who met at least one of the criteria for edoxaban dose reduction, 114 (58.5%) had their doses reduced. Of the remaining 873 patients with available data, 110 (12.6%) patients qualified for a 60 mg dose but were prescribed 30 mg.

There were no clinically significant differences in episodes of MB or CRNMB among the different age groups (aged < 65 years = 0.86% [95% CI 0.10% to 3.07%]; aged ≥65 to <75 years = 1.75% [95% CI 0.70% to 3.56%]; aged ≥75 years = 0.77% [95% CI 0.21% to 1.95%]) and renal function groups (creatinine clearance ≤50 mL/min = 2.11% [95% CI 0.58% to 5.30%]; creatinine clearance >50 mL/min =1.08% [95% CI 0.50% to 2.04%]).

### 3.3 Interruption

Preprocedural interruption (median 2 days; range 1‐6 days) occurred in 781 (67.6%), and 308 patients (26.7%) had a postprocedural interruption (median 3 days, range 1‐29 days). Pre‐ and postprocedural interruption occurred together in 279 (24.2%) of patients. Data on timing of interruption and outcome is summarized in Table 3. See Table 4 for clinical outcomes stratified by EHRA preprocedural recommendations adherence.

**TABLE 3:** Edoxaban interruption and associated outcomes

No edoxaban interruption	# of subjects		MB	MB & CRNMB	All bleeding	Death—anycause		ACS	ATE & cardiac deaths		Clinically relevant events (MB + CRNMB and thrombosis)
	345		0	3 (0.9%)	14 (4.1%)	1 (0.3%)		0	0		3 (0.9%)
Preprocedural interruption: N = 781, mean 1.9 ± 1.08 days. Median 2.0 days, Q1 1.0, Q3 2.0, range 1‐6											
None	374		0	3 (0.8%)	17 (4.5%)	2 (0.5%)		0	0		3 (0.8%)
1 day	358		2 (0.6%)	4 (1.1%)	9 (2.5%)	1 (0.3%)		0	1 (0.3%)		5 (1.4%)
2 days	251		1 (0.4%)	3 (1.2%)	8 (3.2%)	0		0	2 (0.8%)		5 (2.0%)
3 days	111		2 (1.8%)	2 (1.8%)	11 (9.9%)	0		1 (0.9%)	0		3 (2.7%)
≥4 days	61		0	1 (1. 6%)	4 (6.6%)	2 (3.3%)			3 (4.9%)		4 (6.6%)
Postprocedural interruption: N = 308, mean 6.6 ± 8.24 days. Median 3.0 days, Q1 1.0, Q3 8.0, range 1‐29											
None	847		1 (0.1%)	4 (0.5%)	24 (2.8%)	1 (0.1%)		0	1 (0.1%)		5 (0.6%)
1 day	112		0	2 (1.8%)	6 (5.4%)	0		1 (0.9%)	0		3 (2.7%)
2 days	36		0	1 (2.8%)	3 (8.3%)	2 (5.6%)		0	1 (2.8%)		2 (5.6%)
3 days	22		0	0	1 (4.5%)	0		0	0		0
≥4 days	138		4 (2.9%)	6 (4.3%)	15 (10.9%)	2 (1.4%)		0	4 (2.9%)		10 (7.2%)

Abbreviations: ACS, acute coronary syndrome; ATE, acute thromboembolic event; CRNMB, clinically relevant non‐major bleeding; CV, cardiovascular; EHRA, European Heart Rhythm Association; MB, major bleeding.

**TABLE 4:** Clinical outcomes stratified by EHRA preprocedural recommendation adherence

		Minor risk procedures (EHRA recommendation: no interruption)	Low risk procedures (EHRA recommendation: ≥24 hours interruption)	High risk procedures (EHRA recommendation: ≥48 hours interruption)	Total patients
		N = 294	N = 581	N = 280	N = 1155
EMIT pts with preinterruption time according to EHRA recommendation	N	224/294 (76.2%)	320/581 (55.1%)	127/280 (45.4%)	671/1155 (58.1%)
	MB/CRNMB	1 (0.4%)	3 (0.9%)	4 (3.1%)	8 (1.2%)
	ATE/ACS/cardiac death	0 (0%)	2 (0.6%)	1 (0.8%)	3 (0.4%)
EMIT pts with preinterruption time shorter than EHRA recommendation	N	NA	212/581 (36.5%)	123/280 (43.9%)	335/1155 (29.0%)
	MB/CRNMB	NA	1 (0.5%)	2 (1.6%)	3 (0.9%)
	ATE/ACS/cardiac death	NA	0 (0%)	0 (0%)	0 (0%)
EMIT pts with preinterruption time ≥ 24 hours longer than EHRA recommendation	N	70/294 (23.8%)	49/581 (8.4%)	30/280 (10.7%)	149/1155 (12.9%)
	MB/CRNMB	0 (0%)	2 (4.1%)	0 (0%)	2 (1.3%)
	ATE/ACS/cardiac death	1 (1.4%)	0 (0%)	3 (10.0%)	4 (2.5%)

Abbreviations: ACS, acute coronary syndrome; ATE, acute thromboembolic event; CRNMB, clinically relevant non‐major bleeding; EHRA, European Heart Rhythm Association; MB, major bleeding.

Preprocedural interruption of edoxaban demonstrated some differences between clinical practice and EHRA Guidelines. In the 294 patients undergoing minor risk procedures, where guidelines recommend no interruption, 224 patients (76%) had no preprocedural interruption, and 70 (24%) had an interruption lasting longer than recommended. In EHRA low bleeding risk procedures, where EHRA recommends 24‐48 hours interruption, 212 of 581 (36%) of patients had an interruption shorter than recommended, and 49 (8.4%) had an interruption longer than recommended. In high bleeding risk procedures, where a minimal interruption of 48 hours is recommended, 123 of 280 (44%) gad a shorter interruption than recommended, and 30 (11%) had an interruption longer than 72 hours. See Table 4 for details.

## 4 DISCUSSION

### 4.1 Outcomes

We report on the risks of bleeding and thromboembolic events from the first prospective observational multicenter multinational study on periprocedural management and outcomes of 1155 unselected procedures in patients managed with edoxaban therapy. A wide range of medical specialties was procedurally represented, and 23 clinically relevant complications were reported. Baseline characteristics were similar to those from other non‐observational studies.^10,11,20^ This investigation complements the PAUSE study,^11^ by describing outcomes associated with edoxaban, which were not investigated in the PAUSE study. With inclusion of any patient or procedure, the outcomes of a wide range of procedures were evaluated. Five cases of MB (0.4%) and eight of CRNMB (0.7%) were documented. There were five (0.4%) deaths from any cause, two of which were cardiovascular deaths, and six ATE including one ACS. Bleeding incidence was low, even in EHRA classified high risk procedures, being 13/1155 (1.1%) overall for MB/CRNMB and 6/280 (2.1%) in high risk patients.

The majority of patients, 877 (75.9%), received the recommended edoxaban dose based upon label requirements and recorded nine episodes of MB or CRNMB (1.0%), Five ATE and three deaths (0.9%), One hundred and ten patients (9.5%) were on non‐recommended 30 mg dosing, with one episode of MB or CRNMB (0.9%) and a 1.8% rate of ATE/ACS / mortality. Eighty‐one patients (7.0%) were on non‐recommended 60 mg dosing, with three episodes of MB or CRNMB (3.7%) and a 2.5% rate of ATE/ACS / mortality. Use of doses greater than recommended was associated with the highest rates of both significant bleeding and thromboembolic complications or death. Only 114 of 195 (58.5%) of patients had doses reduced to the recommended 30 mg, in spite of the presence of at least one of the dose reduction factors.

In the majority of procedures investigators decided to interrupt edoxaban preprocedurally (781 of 1155 patients, 67.6%). Edoxaban therapy was continued without any interruption during the periprocedural period in 345/1155 (29.9%) patients and 29/1155 (2.5%) patients had only postprocedural interruption. Of the 294 patients undergoing procedures characterized as minor risk, for whom no interruption is suggested, therapy was interrupted preprocedurally in 70 (23.8%). In the low and high‐risk groups, in whom interruption might have been expected, edoxaban treatment was interrupted preprocedurally as recommended in 447 (51.9%) patients. These results indicate that routine clinical practice did not always follow guideline recommendations, but, nonetheless, resulted in low rates of bleeding and ischemic complications in patients receiving edoxaban.

### 4.2 Comparison with published data

The first data on the periprocedural outcomes of NOAC were derived as sub‐analyses from the large Phase III studies of these compounds. In a sub‐study of the ARISTOTLE trial,^10^ the periprocedural management of apixaban was associated with an incidence of MB of 1.62% and of stroke or systemic embolism of 0.35% at 30 days. In 37.5% of patients apixaban treatment was not interrupted periprocedurally. (“Interruption” was defined as no interruption of any dose or interruption on the same day of the operation, which allowed two doses of apixaban to be omitted, and is different from the definition of interruption in the present study.) A sub‐ analysis of the RE‐LY study^22^ found an incidence of major periprocedural bleeding from 7 days before until 30 days of 3.8% for the 110 mg and 5.1% for the 150 mg two times daily regimens. Dabigatran‐treated patients received the last dose a median of 49 (interquartile range, 35‐85) hours before the procedure. The incidence of a composite of thrombotic events ranged from 1.2% for the lower and 1.5% for the higher dabigatran dose. In a post‐hoc analysis of ENGAGE AF‐TIMI,^14^ in the group of patients with periprocedural anticoagulant interruption, major periprocedural bleeding occurred in warfarin treated patients (1.0%), high dose edoxaban (1.2%) and low dose edoxaban (1.1%). Stroke or systemic embolism rates for the same groups were 0.6%, 0.5%, and 0.9%. In the group of patients with no periprocedural anticoagulant interruption, major periprocedural bleeding occurred in warfarin treated patients (3.6%), high dose edoxaban (2.6%) and low dose edoxaban (2.4%). Stroke or systemic embolism rates for the same groups were 1.1%, 0.7%, and 0.9%.Our data on edoxaban show a similar safety signal in terms of MB episodes.

In an evaluation of periprocedural management with NOAC, the DRESDEN registry^20^ reported from 863 procedures predominantly treated with rivaroxaban, that major postprocedural bleeding and cardiovascular events occurred at 30 days in 1.2% and in 1.0% of patients, respectively. The interruption of edoxaban prior to cardiovascular procedures has been studied in Phase III studies specifically addressing cardiovascular procedures such as PCI and AF ablation^4^, ^5^, ^6^ Data on the safety of a standardized perioperative protocol on NOAC management strategy (the NOAC used included apixaban, dabigatran, and rivaroxaban but not edoxaban) has recently become available from the PAUSE study,^11^ which included 3007 procedures. This study tested a predefined interruption protocol and mandated interruption of oral anticoagulation for at least 1 day and, therefore, could include only elective procedures. These criteria are distinct from EMIT AF/VTE in which there was no predetermined management protocol and where 4.3% of procedures were emergency procedures. At 30 days the PAUSE study reported postprocedural rates of MB of 1.35% for apixaban, 0.90% for dabigatran, and 1.85% for rivaroxaban cohorts with arterial thromboembolism occurring in 0.16%, 0.6%, and 0.37%, respectively.

Overall, these event rates in PAUSE appear to be similar to those documented in EMIT, even though a direct comparison of the bleeding and ischemic risks of both study populations has its limitations. PAUSE, for example, included a higher percentage of high bleeding‐risk procedures (33.5%) than EMIT (24.2%), but only EMIT included emergency procedures.

As EMIT is a study of unselected patients taking edoxaban, VTE was the indication for edoxaban administration in 8.6% of the patients. However, the low incidence of events did not allow us to distinguish between AF and VTE patients. PAUSE^11^ was a pure AF patient trial that excluded patients with VTE as the indication for oral anticoagulation.

Although there are design differences between EMIT, DRESDEN,^20^ and PAUSE,^11^ all three studies suggest to physicians that for the majority of patients short‐term interruption is the preferred approach in periprocedural anticoagulation management in order to minimize hemorrhagic or thromboembolic complications.

### 4.3 Limitations of study

One limitation of this study is lack of a comparator arm and its restriction to one NOAC, since differences exist between the various NOACs. Further, no attempt was made to evaluate the impact of geographical location or ethnicity.

However, given the plethora of diagnostic and interventional procedures, the conduct of a global study/registry that would address the above limitations is logistically and economically not feasible. The EMIT data set, being unique in capturing observational rather than prescriptive management of patients on edoxaban in real life setting, complements the data from DRESDEN^20^ and PAUSE.^11^ The three studies together indicate that oral anticoagulation, when managed carefully, is associated with a low incidence of major hemorrhagic and significant ischemic events.

Observational studies often do not provide the same level of data quality as controlled studies. However, patients were provided with memory aids to support data collection (which were reviewed for completeness at the end of the procedural follow‐up), critical data elements were reviewed at the patient level, and all critical events were centrally adjudicated. Offsetting any loss in quality because of missing data or lack of mechanisms to support compliance is the ability of an observational study to accurately reflect current clinical practice without external influence. Rather than adhering to a study protocol defined treatment regimen, physicians were free to follow their understanding of best practices for a given patient.

## 5 CONCLUSIONS

In patients taking edoxaban for an approved indication, the periprocedural occurrence of MB and CRNMB as safety measures, and ATE and ACS as markers of efficacy, were low in the overall study and all patient subgroups. EMIT AF/VTE documents periprocedural edoxaban management, with short interruption times as a safe and effective approach that prevents adverse outcomes.

Further study is required to better understand the risk factors in those few patients who develop bleeding or ischemic complications.

## CONFLICT OF INTEREST

Paolo Colonna reports grants and personal fees from Daiichi Sankyo Europe and personal fees from Daiichi Sankyo Italy during the conduct of the study; personal fees from Boehringer Ingelheim, BayerAG, and Pfizer/BMS; and nonfinancial support from the European Society of Cardiology (ESC) and the Italian Cardiology Association (ANMCO) outside the submitted work. Christian von Heymann reports grants and personal fees from Daiichi Sankyo Europe and Daiichi Sankyo Germany during the conduct of the study; personal fees from Boehringer Ingelheim, Bayer AG, Pfizer GmbH, CSL Behring, NovoNordisk Pharma, Mitsubishi Pharma, Ferring GmbH, Biotest GmbH, and Leo Pharma GmbH outside the submitted work; that he was mandated from the German Society of Anaesthesiology and lntensive Care Medicine (DGAI) to write the German Guideline on Preoperative Anaemia (published in April 2018); was part of the writing group of the Patient Blood Management Guideline in cardiac surgery on behalf of the European Society of Cardiothoracic Anaesthesiologists (EACTA) in conjunction with the European Society of Cardiothoracic Surgery (EACTS) (published in September 2017); and was mandated to take part in the writing group of the guideline on the Diagnostics and Treatment of Peripartum Haemorrhage of the Deutsche Gesellschaft für Gynäkologie und Geburtshilfe (DGGG) (published in March 2016). Amparo Santamaria reports nothing to disclose. Manish Saxena reports grants and personal fees from Daiichi Sankyo Europe and personal fees from Daiichi Sankyo UK during the conduct of the study. Thomas Vanassche reports grants and personal fees from Daiichi Sankyo Europe; and personal fees from Daiichi Sankyo Be, Bayer, 367 Leo Pharma, and Boehringer Ingelheim during the conduct of the study. Petra Laeis, Cathy Chen, and Martin Unverdorben are employees of Daiichi Sankyo. Robert Wilkins reports personal fees from Daiichi Sankyo, Inc., during the conduct of the study.

## Supporting information


**Supplementary Figure S1** Procedures by medical specialty and outcomes

**Supplementary Table S1** Outcomes categorized by EHRA bleeding risk category, HAS‐BLED and CHA_2_DS_2_VASc risk scores and outcomes
