# Supplementary figures and images for: Routine clinical practice in the periprocedural management of edoxaban therapy is associated with low risk of bleeding and thromboembolic complications: The prospective, observational, and multinational EMIT‐AF/VTE study
Source: Clin Cardiol. 2020 May 14;43(7):769–80. doi: 10.1002/clc.23379 (PMC7368298; doi:10.1002/clc.23379)

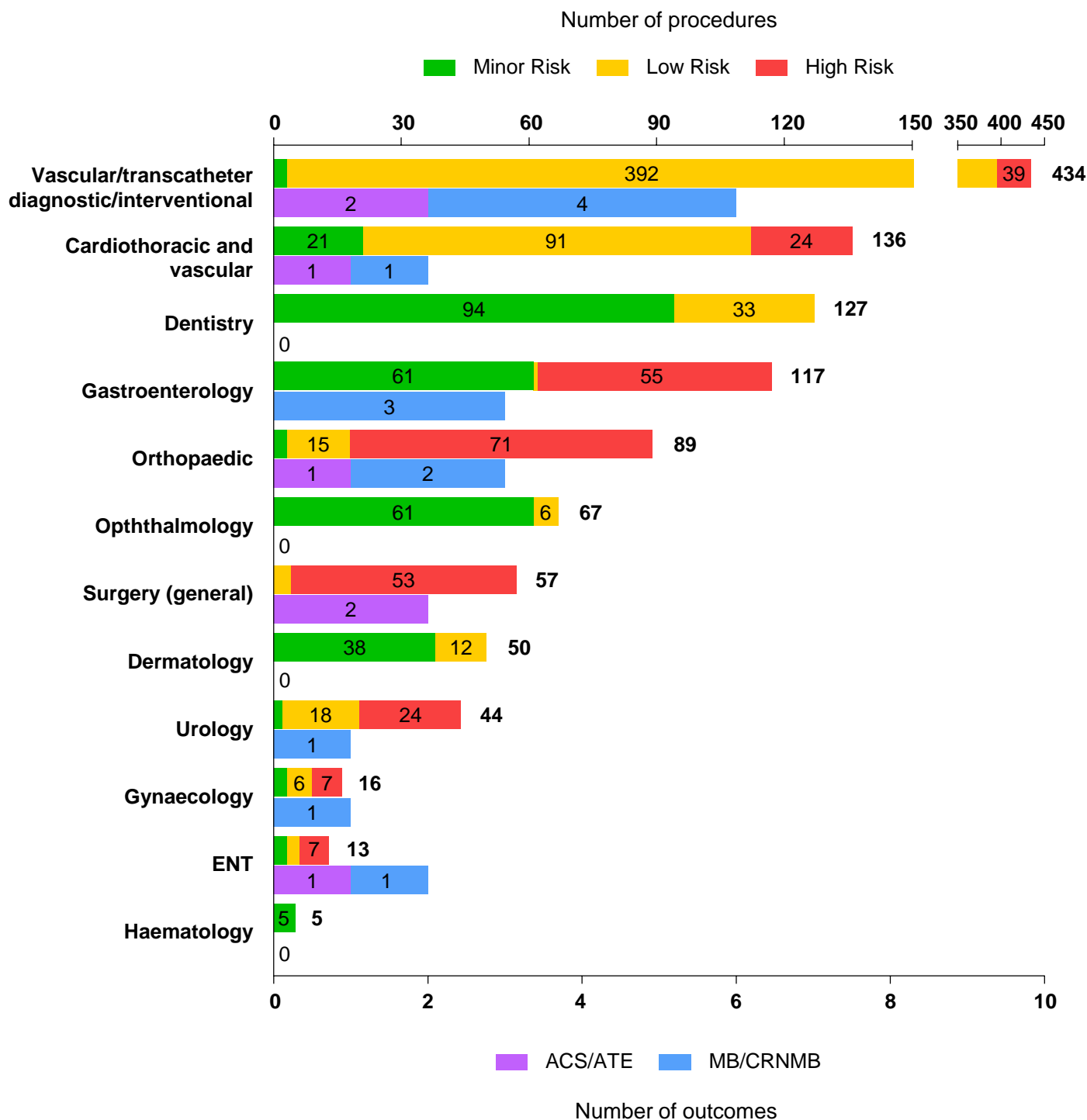

Supplement: Supplementary file 1 — Supplementary Figure S1 Procedures by medical specialty and outcomes [file CLC-43-769-s001.pdf]
