# Supplementary material for: Routine clinical practice in the periprocedural management of edoxaban therapy is associated with low risk of bleeding and thromboembolic complications: The prospective, observational, and multinational EMIT‐AF/VTE study
Source: Clin Cardiol. 2020 May 14;43(7):769–80. doi: 10.1002/clc.23379 (PMC7368298; doi:10.1002/clc.23379)
Supplement: Supplementary file 2 — Supplementary Table S1 Outcomes categorized by EHRA bleeding risk category, HAS‐BLED and CHA2DS2VASc risk scores and outcomes [file CLC-43-769-s002.docx]

## **Supplementary Table 1: Outcomes categorized by EHRA Bleeding Risk Category, HAS-BLED and CHA_2_DS_2_VASc Risk Scores and Outcomes**

|  | **EHRA Bleeding Risk**  **n = 1,155** | | |  | **HAS-BLED Score**  **n = 724*** | | |  | **CHA_2_DS_2_VASc Score**  **n = 1,114** | | | |
| --- | --- | --- | --- | --- | --- | --- | --- | --- | --- | --- | --- | --- |
|  | ***Minor*** | ***Low*** | ***High*** |  | ***HAS-BLED Risk Factor Low (0-1 point)*** | ***HAS-BLED Risk Factor Medium (2 points)*** | ***HAS-BLED Risk Factor High (≥3 points)*** |  | ***CHA_2_DS_2_VASc Risk Factor Low (0-1 point)*** | ***CHA_2_DS_2_VASc Risk Factor Moderate (2-4 points)*** | ***CHA_2_DS_2_VASc Risk Factor High (>4 points)*** |  |
| Number of subjects | 294 (25.5%) | 581 (50.3%) | 280 (24.2%) |  | 338 (46.7%) | 237 (32.7%) | 149 (20.6%) |  | 160 (14.4%) | 733 (65.8%) | 222 (19.9%) |  |
| All Bleeding (%, 95% CI) | 9 (3.1%,  1.4%,5.7%) | 24  (4.1%, 2.7%,6.1%) | 16  (5.7%,  3.3%,9.1%) |  | 9  (2.7%,1.2%,5.0%) | 13 (5.5%, 3.0%,9.2%) | 6  (4.0%,  1.5%,8.6%) |  | 6 (3.8%,  1.4%,8.0%) | 29  (4.0%,  2.7%,5.6%) | 10 (4.5%,  2.2%,8.1%) |  |
| MB or CRNMB | 1 (0.3%) | 6 (1.0%) | 6 (2.1%) |  | 1 (0.3%) | 3 (1.3%) | 3 (2.0%) |  | 0 | 12 (1.6%) | 1 (0.5%) |  |
| ATE† | 0 | 2(0.3%) | 4 (1.4%) |  | 0 | 3 (1.3%) | 2 (1.3%) |  | 0 | 5 (0.7%) | 1 (0.5%) |  |
| ACS | 1 (0.3%) | 0 | 0 |  | 0 | 0 | 0 |  | 0 | 1 (0.1%) | 0 |  |
| CV Mortality | 0 | 1 (0.2%) | 1 (0.4%)^ |  | 0 | 0 | 2 (1.3%)^ |  | 0 | 2 (0.3%) | 0 |  |
| All-cause mortality | 1 (0.3%) | 2 (0.3%) | 2 (0.7%) |  | 0 | 1 (0.4%) | 2 (1.3%) |  | 0 | 4 (0.5%) | 0 |  |

EHRA = European Heart Rhythm Association

HAS-BLED = Hypertension, Abnormal renal/liver function, Stroke, Bleeding history or predisposition, Labile international normalized ratio, Elderly, Drugs/alcohol concomitantly score

CHA2DS2-VASc = Congestive heart failure, Hypertension, Age ≥ 75 [doubled], Diabetes, Stroke [doubled]-Vascular disease, Age 65-74 years, and gender [female]) score

MB = Major Bleeding, CRNMB = Clinically Relevant Non-Major Bleeding, ACS = Acute Coronary Syndrome, ATE = Acute Thromboembolic Event, CV = Cardiovascular

*Missing HAS-BLED scores primarily due to missing INR datapoint

†Stroke, TIA, Systemic Embolic Event, VTE, DVT, PE. ATE numbers include cardiac deaths
